# Stationary Atoms in Liquid Metals and Their Role in Solidification Mechanisms

**DOI:** 10.1021/acsnano.5c08201

**Published:** 2025-12-09

**Authors:** Christopher Leist, Sadegh Ghaderzadeh, Emerson C. Kohlrausch, Johannes Biskupek, Luke T. Norman, Ilya Popov, Jesum Alves Fernandes, Ute Kaiser, Elena Besley, Andrei N. Khlobystov

**Affiliations:** 1 Central Facility Materials Science Electron Microscopy, 9189 Ulm University, Ulm 89081, Germany; 2 Institute for Quantum Optics (IQO), 9189 Ulm University, Ulm 89081, Germany; 3 School of Chemistry, 6123 University of Nottingham, Nottingham NG7 2RD, United Kingdom

**Keywords:** nanoparticle, phase transition, melting, graphene, crystal nucleation

## Abstract

According to common understanding, the primary difference between a liquid and a solid metal lies in atomic motion—atoms move rapidly in liquids, while they remain stationary in a solid lattice. The solidification process involves a transition from random atomic motion to an ordered crystalline structure, with nucleation playing a crucial role. However, our research indicates that the boundary between these two phases is not as distinct as previously believed: liquid metal nanoparticles can contain stationary atoms, and the number and positions of these atoms influence the solidification pathway upon cooling. Using spherical and chromatic aberration-corrected high-resolution transmission electron microscopy (HRTEM) at low accelerating voltages, we studied the solidification of platinum, palladium, and gold. We have developed a methodology that enables imaging of metal particles over a wide temperature range, from 20 to 800 °C, without compromising atomic resolution. When a nanoparticle melts, the contrast contribution of the fast-moving atoms vanishes in the HRTEM images, allowing stationary atoms to be visualized through the liquid layer as distinct atomic points of contrast that remain fixed in position on the imaging time scale (1 s or longer). These atoms are pinned at vacancy defect sites on graphene. By conducting HRTEM image contrast analysis during time-series imaging of individual 3–6 nm particles while changing the temperature from 800 to 20 °C, we uncover the mechanisms behind classical crystal nucleation, amorphous solidification, and the formation of supercooled liquid platinum. If the number of stationary platinum atoms is small (approximately fewer than 10) and positioned randomly, liquid-to-crystal nucleation can occur. However, if the number is higher, these stationary atoms can disrupt the crystallization process, particularly if they align along the perimeter of the liquid nanoparticle. We found that liquid nanodroplets, corralled by stationary atoms, remain liquid down to 200–300 °C, which is several hundred degrees below the bulk metal crystallization temperature. In these cases, supercooled liquid metal transforms into a metastable amorphous solid instead of crystallizing. Our results highlight the significance of stationary atoms in liquids, influenced by the local environment, which may hold significant implications for the use of metal nanoparticles on carbon in heterogeneous catalysis and other thermally activated processes.

## Graphical abstract

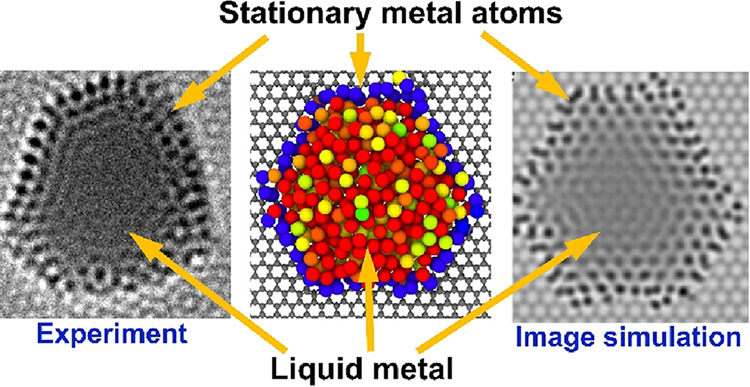

## Introduction

The formation of solids is crucial in various natural phenomena such as mineralization, ice crystallization, and protein fibril folding, as well as technological processes such as metallurgy and pharmacy. Fundamental principles of interatomic bonding and interactions of atoms with the environment regulate this process, often displaying unusual thermodynamics and complex kinetics, different from classical processes.^1–4^ Real-time microscopy methods allow direct observation of nucleation and propagation of solid phases, making them powerful tools for unlocking the mechanisms of crystallization.^5–11^ This has revealed that even the solidification of pure metals - the simplest type in which atoms of the same kind join together to form a lattice - takes place via intricate atomic mechanisms. For instance, the solidification of bismuth, which has been extensively studied by transmission electron microscopy (TEM), demonstrates that the ordering of atoms can start either on the interface of molten metal with the support or on the surface of the molten metal, followed by a one-dimensional preordering of atoms before crystallization.^12–16^ TEM imaging enabled the investigation of early nucleation stages in gold, rhenium, and iron to reveal that there is a critical number of atoms which must come together in a two-step process to initiate a stable crystalline nucleus.^17^

In this work, we use spherical and chromatic aberration-corrected high-resolution TEM (C_c_/C_s_-corrected HRTEM) imaging to visualize atomic dynamics during a fundamental process: the transformation from liquid to solid. We show that within liquid nanoparticles, some metal atoms are bonded to the support, and they remain stationary irrespective of the temperature. The number and spatial distribution of stationary atoms within molten nanoparticles vary based on the local environment, which can be altered by the electron beam. We found that the fraction of stationary atoms plays a crucial role in the solidification process, including the formation of metastable crystal nuclei, and coexistence of solid, amorphous and crystalline phases within the same nanoparticle. Stationary atoms surrounding a liquid particle can lead to a supercooled state of molten metal more than 1000 °C below the bulk solidification temperature. All these processes coexist within the same material and are strongly influenced by local particle environments, as demonstrated by our atomically resolved, continuous observations of the solidification dynamics over a wide temperature range, 20–800 °C.

## Results and Discussion

### Experimental Approach: In Situ Heating with Dual Use of the Electron Beam

We use single-layer graphene (SLG) on a heated TEM grid as a support for metal atoms deposited directly onto it by magnetron sputtering (Step 1, Figure 1a). Metal atoms self-assemble into clusters whose size is controlled by the surface density of metal atoms, the concentration and nature of surface defects, and temperature.^18,19^ Our method does not require the use of any reagents or solvents, thus ensuring that platinum, palladium and gold are studied in their native metallic form in HRTEM experiments.

**Figure 1:**
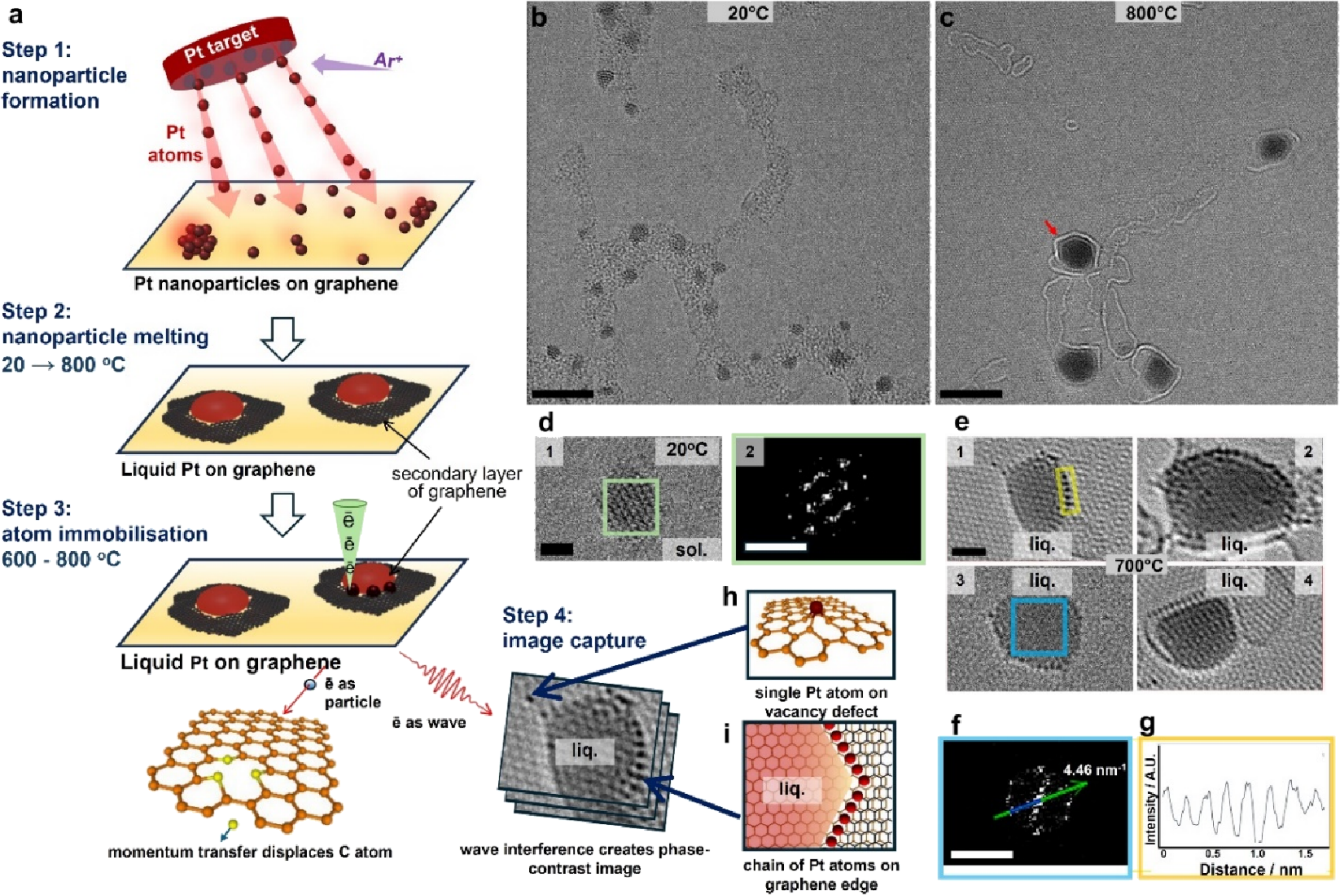
Materials preparation and experimental methodology. (a) An experimental workflow including the direct assembly of metal nanoparticles on graphene (Step 1), followed by melting nanoparticles by in situ heating inside the TEM column, when nanoparticles become liquid and the adventitious carbon on graphene anneals into islands of the second layer of graphene to which the liquid nanoparticles are adhered (Step 2). The electron beam of TEM serves a dual purpose as a source of energy transferred primarily to the carbon atoms of graphene (Step 3) and an imaging tool, creating phase-contrast images at the same time (Step 4). The vacancy defects created in Step 3 entrap single Pt atoms (h), while open edges of the second layer of graphene - chains of Pt atoms (i). HRTEM images of Pt nanoparticles on graphene: (b) at room temperature and (c) at 800 °C (carbon surrounding liquid Pt is highlighted by defocus conditions). (d) A zoomed-in image of an individual Pt nanoparticle at room temperature, where the positions of atom columns are clearly visible, and the FFT of the area in the green square showing the presence of the fcc Pt lattice. (e) Zoomed-in images of molten Pt nanoparticles at 800 °C where individual Pt atoms are visible at graphene vacancies or edges, while molten nanoparticles show uniform contrast of liquid metal, making the graphene lattice visible, and a convex meniscus, confirming the liquid state. (f) A FFT of a liquid Pt nanoparticle (area in the blue square) showing no features corresponding to the metal lattice and confirming the liquid state. The liquid Pt nanoparticle becomes transparent, so that the FFT corresponds to the graphene lattice underneath. (g) Intensity line profile of a chain of Pt atoms along the edge of a graphene layer (orange box in panel e1) demonstrating a periodic spacing between the stationary metal atoms (scale bars: (b, c) 5 nm, (d1, e) 1 nm, (d2, f) 10 nm^–1^).

By utilizing microelectro-mechanical system (MEMS) heated chip technology in combination with the high thermal conductivity of graphene, we can control the temperature of the sample within a range of 20–800 °C. Once the temperature has stabilized and the sample’s drift caused by thermal expansion or contraction has subsided, atomic resolution in the images can be maintained (Step 2, Figure 1a). Due to graphene’s thinness and high thermal conductivity, beam-induced heating is not a concern in the sample.^20,21^ As the temperature changes, we use C_c_/C_s_-corrected HRTEM imaging at 60 or 80 keV electron beam to monitor transformations in individual nanoparticles.

The dual nature of electrons also allows us to use their wave nature to generate images through the phase contrast mechanism (Step 4, Figure 1a), as well as harness their momentum, enabling them to act as projectiles on carbon atoms in graphene. This interaction, termed the direct knock-on (DKO) effect, can displace carbon atoms from the graphene lattice and create additional vacancies (Step 3, Figure 1a). The 80 keV electron beam can transfer kinetic energy up to 15.8 eV to graphene’s carbon atoms (eq 1, where θ is the electron scattering angle, electron beam energy *E*, masses of the atom and the electron *m*_*n*_ and *m*_*e*_, respectively, and speed of light *c*), which is considered to be below the displacement threshold of ca. 17 eV.

$$E_{T}\left(\theta \right)=\frac{2m_{n}E\left(E+2m_{e}c^{2}\right)}{{\left(m_{n}+m_{e}\right)}^{2}c^{2}+2m_{n}E}sin^{2}\left(\frac{\theta }{2}\right)=E_{T\text{\_}\max}sin^{2}\left(\frac{\theta }{2}\right)$$ 1

However, in our experiments, Pt and other metals act as catalysts, lowering the energy barrier for carbon displacement,^22^ which is corroborated by our density functional theory (DFT) calculations (Figures S1 and S2). Vacancy defects in graphene serve as effective binding sites for metal atoms through the formation of covalent M-C bonds. Consequently, when we irradiate molten metal nanoparticles, as more vacancy defects in graphene are generated, more metal atoms become bonded to the graphene. The rate of this process *r* is governed by the rate of DKO, which is proportional to the electron beam flux *j* (eq 2, where σ_*d*_ is the displacement cross section of C atom from the graphene and *[C]* is the areal density of C atoms).^23^

$$r=\sigma_{d}j\left[C\right]$$ 2

Our measurements indicate that significant changes in the number of stationary metal atoms in liquid Pt nanoparticles occur only at very high flux values, approximately *j* ∼ 10^9^ electrons/(nm^2^ s). However, the nature of the metal plays a significant role; for Au, even this flux is insufficient, whereas for Pd, a moderate flux of ∼ 10^6^ electrons/(nm^2^s) triggers fast changes that are difficult to follow in real time. In molten Pt, the high flux enables us to control the number and location of stationary metal atoms, bonded to vacancy defects in graphene, by irradiating a selected liquid nanoparticle with a condensed electron beam (Step 3, Figure 1a). This allows us to study the impact of the number of stationary atoms on the solidification of Pt as we subsequently lower the temperature of the sample, capturing HRTEM images of specific nanoparticles step by step (Step 4, Figure 1a).

### Observing and Manipulating the Environment around Liquid Nanoparticles Using an Electron Beam

C_c_/C_s_-corrected HRTEM imaging reveals that at room temperature, metal atoms on SLG aggregate into 2–3 nm nanoparticles next to the adventitious amorphous carbon present on the graphene (Figure 1b). Metal atoms are clearly visible on graphene due to the low contrast of the underlying carbon, often assembling into a lattice that resembles the face-centered cubic (fcc) lattice of bulk Pt (Figure 1d). Theoretical modeling of Pt nanoparticles predicts that Pt(111) on graphene is the most stable.^2,24^

Heating to 800 °C causes the sharp atomic contrast in solid nanoparticles to transition to a smooth, diffuse contrast (Figure 1c and Figure S3), and smooth convex menisci, expected for a liquid nanodroplet, indicating melting (Figure 1e). FFT reflections associated with Pt metal lattice disappear, with the only lattice contrast corresponding to underlying graphene, which becomes visible through the layer of liquid metal (Figure 1f). However, we found that the atomic contrast of some isolated Pt atoms remains sharp and unaffected by heat, suggesting that their positions remain constant for at least the period of image capture (approximately 1 s per frame) or longer, as they are bound to vacancy defects (Figures 1e and 1h). In some instances, the second layer of graphene, formed from annealed amorphous carbon, wraps around the nanodroplets, maintaining a 0.4 nm gap (Figure 1e4 and Figure S4). Similar behaviors were noted for molten Pd and Au nanoparticles (Figures S5 and S6).

Our time-series HRTEM images reveal carbon atom displacement in contact with Pt, resulting in vacancy defects in graphene that increase with increasing electron beam flux, as expected from eq 2 (Section S1, Supporting Information file), consistent with other metals.^25–27,22^ When defects are located along the zigzag edge of the second layer of graphene surrounding a molten Pt particle, the stationary platinum atoms line up into a chain separated from each other by about 0.25 nm (orange box in Figures 1e and 1g). We demonstrated that irradiation of an individual molten particle with a high flux of the 80 keV electron beam, by condensing the beam onto it for several seconds (Figure 2a), can lead to the liquid nanoparticle becoming surrounded with a ring of pinned Pt atoms (Figure 2b). The particle can be solidified entirely after additional high-flux irradiation (Figure S7). Indeed, liquid nanoparticles can be solidified in a single step by a high-flux electron beam irradiation, transforming them directly into a crystalline phase (Figure 2c), without changing their temperature. This electron beam-promoted solidification takes place in various-sized molten Pt nanoparticles, which form fcc lattices with different orientations (Figure S8). Notably, if the second graphene layer wraps tightly around the nanoparticle, without open edges, the nanoparticle can remain liquid under high electron irradiation (Figure S4). Our observations indicate that controlling the environment around molten Pt nanoparticles by the electron beam enables the effective manipulation of their state, unlike other metals such as palladium or gold, which are more challenging to manage, as detailed later. For example, molten Pd nanoparticles solidify within seconds under a moderate electron flux (Figure S5), while Au nanoparticles stay liquid even under a high flux (Figure S6). Thus, we selected Pt for further dynamic studies due to its more controllable behavior.

**Figure 2:**
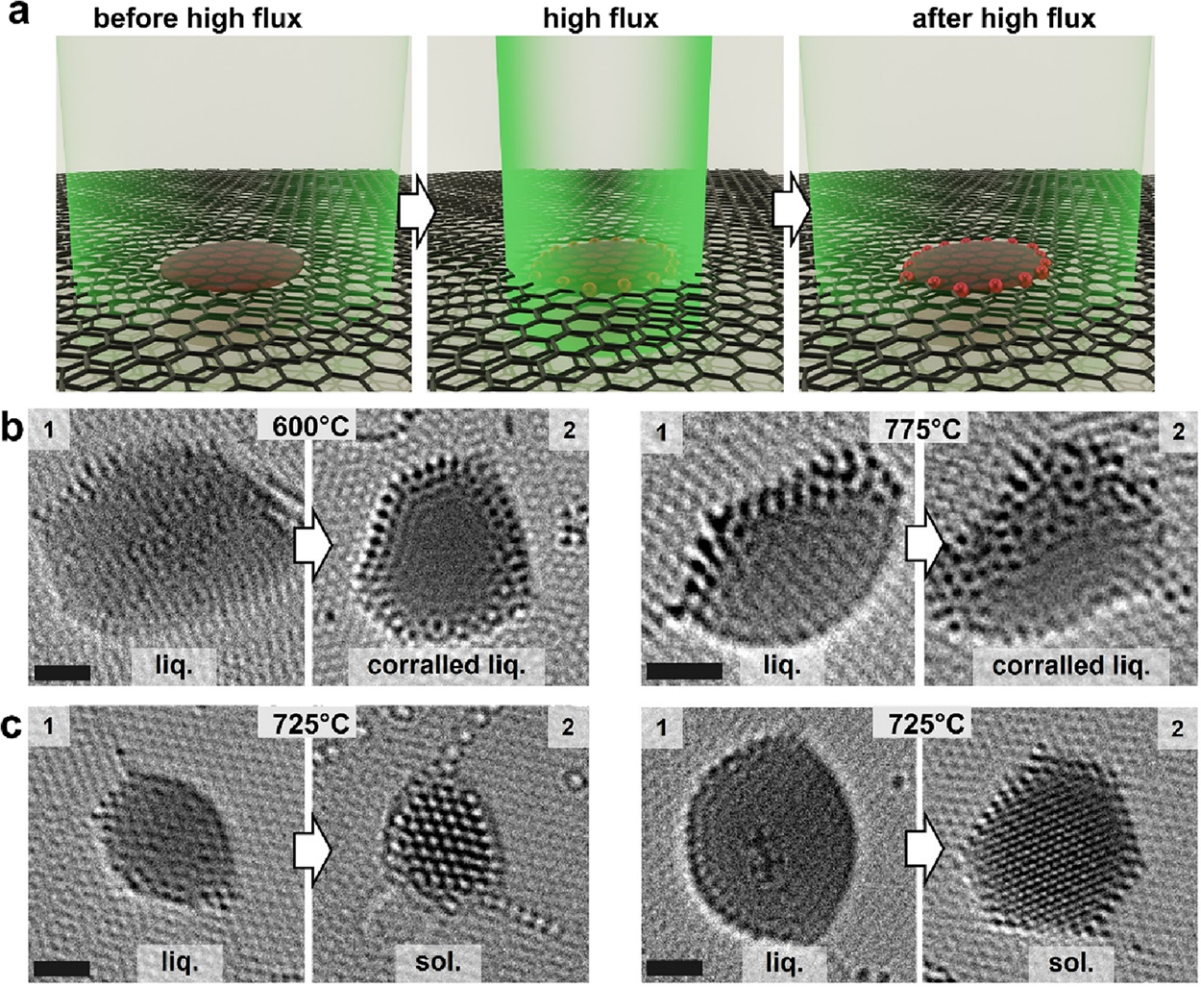
High-flux electron beam controls the number of stationary atoms in liquid nanoparticles by pinning of single Pt atoms to vacancies in graphene (constant temperature). (a) Schematics illustrating high-flux electron beam treatment with 10^8^ e^–^/(nm^2^s) on liquid Pt nanoparticles, and imaging at lower fluxes of 10^6^ e^–^/(nm^2^s) before (left) and after (right) the treatment. The electron beam is illustrated with a green color and stationary atoms in red. HRTEM images of liquid nanoparticles at constant temperature (as annotated on images), before and after high-flux electron beam irradiation, leading to (b) corralled liquid or (c) solid nanoparticles. High-flux irradiation modifies the edges of graphene, affecting the shapes of particles (scale bars: 1 nm). The stationary atoms bonded to the vacancy defects in the basal plane of graphene are effectively immobilized on the time scale of image acquisition, while those bonded to the open edge of the second layer of graphene can undergo occasional hopping from one position to another.

### Studying the Impact of Stationary Atoms on the Dynamics of Liquid Nanoparticle Solidification during Cooling

Using MEMS heated chip technology, we lowered the temperature in steps from 800 to 20 °C while monitoring changes in nanoparticles using HRTEM. Surprisingly, the solidification dynamics of molten Pt exhibited considerable variability, even within the same field of view, resulting in different solidification temperatures and mechanisms, influenced by the local environment (Figures 3a-c). Solidification temperatures, in general, were lower than melting, consistent with the hysteresis predicted by the embedded atom method (EAM) calculations (Figure 3a, inset). In a simple case of homogeneous nucleation, the crystalline phase coexists with the liquid within the same nanoparticle. This metastable state experiences dissolution and recrystallization over minutes, with a critical nucleus size of about 2 nm (Figure 3d). Stationary atoms in the liquid facilitate crystallization from the center, with crystal planes gradually extending throughout the nanoparticle (Figure 3d and Figure S9). However, molten nanoparticles corralled with a ring of stationary Pt atoms behave differently. While uncorralled Pt nanoparticles crystallize around 500 °C, as indicated by the emergence of Pt fcc lattice contrast (Figure 3b), corralled ones remain liquid down to much lower temperatures, fluctuating between liquid and amorphous solid states (Figures 3c, e). Notably, the cores of the corralled nanoparticles remain in a supercooled liquid state even at 350 °C, as indicated by the absence of clear atomic Pt contrast, solidifying as amorphous only at around 200 °C (Figure 4a and Figures S10 and S11). This amorphous solid phase can transform to a crystalline state spontaneously or upon stimulation by electron beam irradiation, causing strain and fracture in the underlying graphene (Figure 4a, last frame). However, larger corralled nanoparticles appear to be able to sustain both crystalline and amorphous phases upon solidification, without straining the graphene (Figure 4b, right frame).

**Figure 3:**
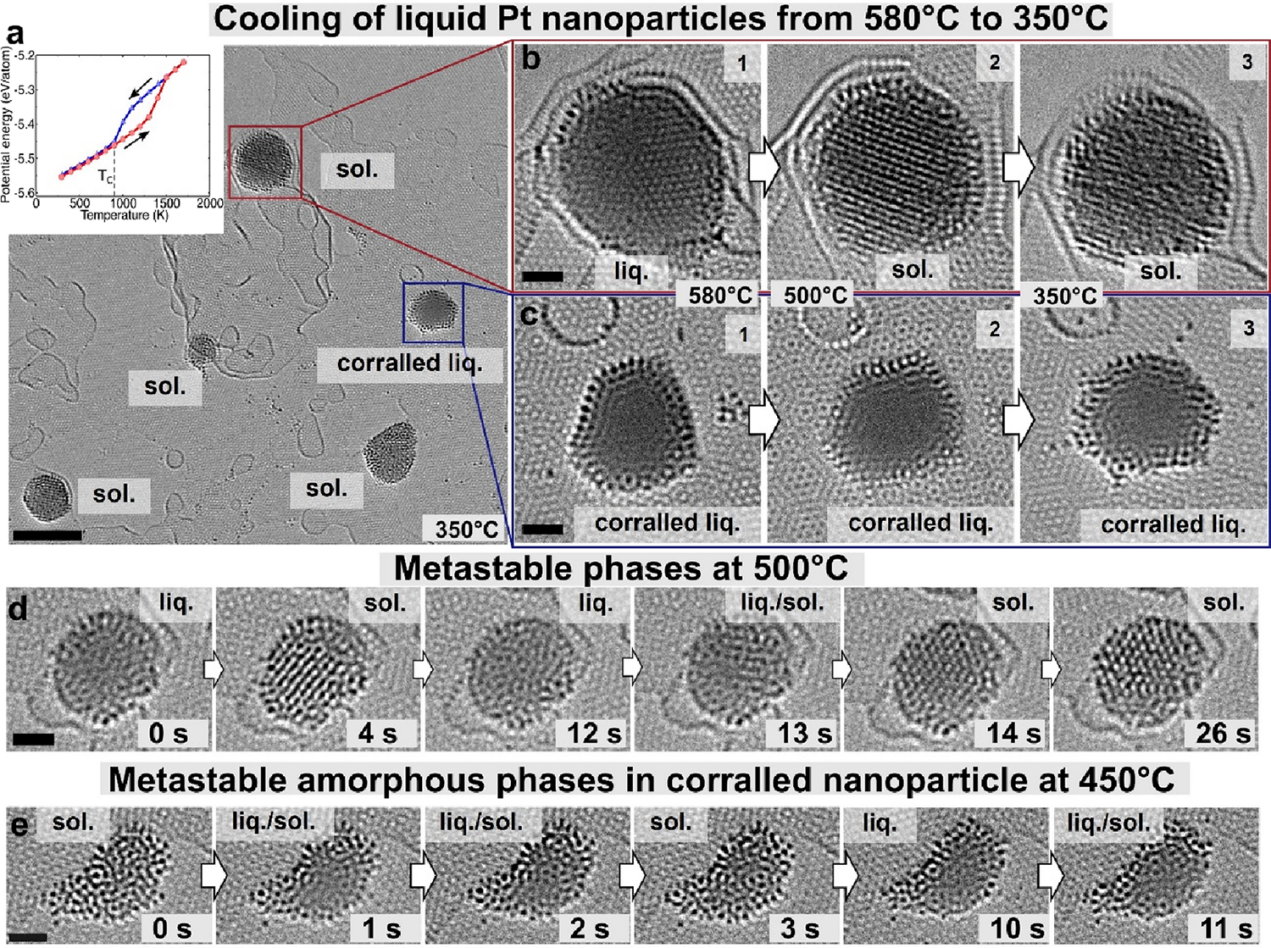
Behaviors of corralled and uncorralled liquid Pt nanoparticles upon cooling. (a) A large field of view showing several Pt nanoparticles on graphene that have been cooled down to 350 °C from 800 °C. All nanoparticles are crystalline solids at 350 °C, apart from one that was irradiated with a high flux electron beam in the liquid state, prior to cooling (blue frame). The inset shows melting (red) and solidification (blue) plots of the potential energy of a free-standing 5.8 nm nanoparticle as a function of temperature calculated using the embedded atom method (EAM) interatomic potential. (b) Stages of solidification of an uncorralled liquid Pt which becomes a crystalline solid when cooled from 580 to 500 °C, (c) in contrast, a corralled Pt nanoparticle remains liquid at 500 °C and even at 350 °C. Both particles are located in the same field of view (a), thus experiencing the same temperature. (d) Time series of images at 500 °C showing metastable crystalline phases emerge and disappear in the same liquid nanoparticle that contained a small number of stationary atoms at the start (0 s) liquid, (4 s) crystalline, (12 s) liquid, (13 s) mixed liquid and crystalline, (14 s) crystalline, (26 s) crystalline phases. (e) A time series of images of a corralled nanoparticle at 450 °C showing fluctuation of liquid and amorphous solid phases, which indicate a high barrier for crystallization in the corralled state (scale bars: (a) 5 nm, (b, c, d and e) 1 nm).

**Figure 4:**
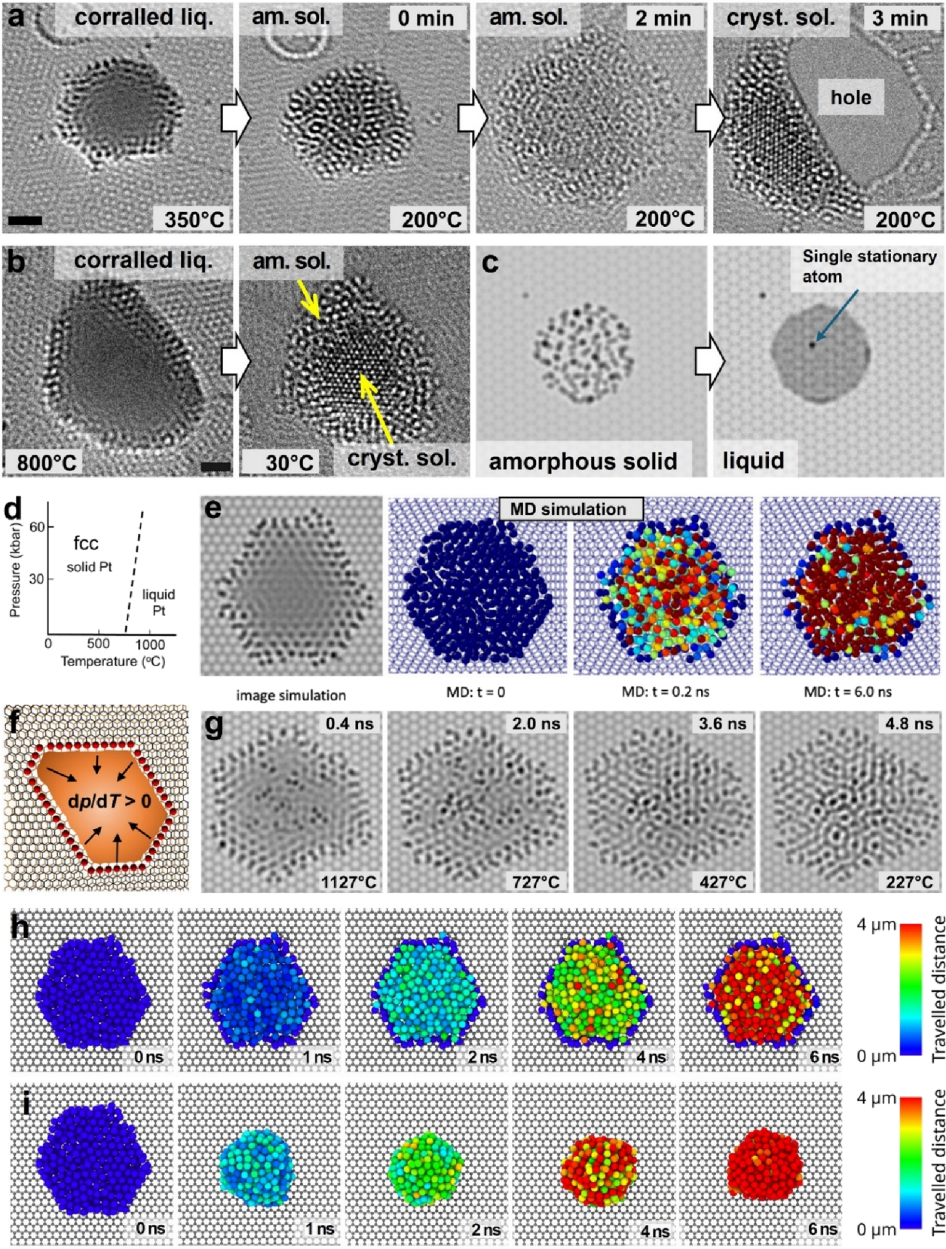
Solidification of corralled liquid Pt nanoparticles. HRTEM images depict the solidification mechanism of corralled liquid Pt nanoparticles. (a) A small supercooled corralled liquid nanoparticle at 350 °C solidifies into an amorphous phase when cooled to 200 °C, spreading on graphene and later transforming into a crystalline phase, which causes strain-induced tearing of the graphene. (b) Larger corralled liquid Pt nanoparticle directly transforms into a crystalline phase at the core surrounded by an amorphous shell. (c) Simulated HRTEM image of amorphous solid (left) and liquid (right) Pt nanoparticles on graphene; in the liquid state, the nanoparticle is transparent to the electron beam, revealing the graphene lattice. (d) A schematic phase diagram for Pt at the nanoscale, taking into account the Gibbs–Thomson effect. (f) A schematic diagram illustrates negative pressure during crystallization as nanoparticle volume decreases, pulling atoms inward, offset by Pt–C bonding and tensile strength of graphene. (e) MD calculation of the temporal evolution of a corralled liquid Pt nanodroplet showing the degree of translational motion of Pt atoms over time at a temperature of 1027 °C. The color of Pt atoms in the model indicates the displacements from their original coordinates: red - large displacement, blue - small displacement. An HRTEM image (leftmost) simulated from the MD model. (g) Time series of HRTEM images simulated from selected MD models illustrating the solidification process from 1127 to 27 °C, in 100 °C steps every 0.5 ns (full dynamic is shown in Video S8). MD calculations comparing the temporal dynamics of a corralled (h) and free (i) liquid Pt at 1027 °C, from the same starting point (first frame). The colors represent distances traveled by Pt atoms, rather than displacements. Atomic displacements were included in the traveled distance every 2 ps, except when they fell below a 2.5 Å threshold, in order to discount vibrations (full dynamic is shown in corresponding Supplementary Videos) ((a and b) scale bars: 1 nm).

## Discussion of Observations

In our experiments, we have access to two stimuli: temperature to trigger the phase transition in nanoparticles and a high-flux 80 keV electron beam (when the beam is converging on a single nanoparticle, reaching >10^8^ e^–^/(nm^2^s)) to modify the local environment around the nanoparticle. At a moderate electron beam flux of 10^6^ e^–^/(nm^2^s), typically used for HRTEM imaging, we can observe the melting and solidification of nanoparticles unperturbed by the electron beam. During heating to 800 °C, all nanoparticles attach to an adlayer of carbon adsorbed on SLG (formed from adventitious amorphous carbon), while single metal atoms become embedded within vacancy defects, as can be expected from strong metal–carbon covalent bonding.^28–30^ When a nanoparticle is solid, metal atoms form a lattice that exhibits a strong HRTEM image contrast for all three metals,^31^ which completely obscures the graphene lattice (Figures 1b and 1d). However, when the temperature rises above 600 °C, the metal contrast blurs out, the nanoparticle becomes transparent, and the underlying graphene lattice becomes visible, providing a clear distinction between the solid and liquid states (Figures 1c, 1e for Pt, Figures S5 and S6 for Pd and Au, respectively). The observed transition in the HRTEM image can be explained by the high value of self-diffusion coefficient *D* of liquid metals (0.5·10^10^ nm^2^/s for Pt, for example) (1), so that the square root of the mean squared displacement <Δ*r* ^2^ *>* of the metal atom during the image capture time *t* (ca. 1 s; eq 3) is significantly greater than the size of the molten nanoparticle. As a result, on the time scale of HRTEM imaging, the atomic contrast of metal is smeared out.

$$size\;of\;nanoparticle\ll \left\langle \Delta r^{2}\right\rangle =2Dt$$ 3

To distinguish randomly localized and delocalized (mobile) atoms in an amorphous solid and liquid nanoparticle, respectively, we performed HRTEM image simulations using stationary Pt atoms randomly positioned or Pt atoms delocalized within the volume of a nanoparticle (Figure 4c left and right frames, respectively). In remarkable agreement with the experiments (Figure 1e), simulated images for liquid metal demonstrate that the graphene lattice, as well as individual stationary Pt atoms, can be clearly observed by HRTEM through a layer of liquid metal (Figure 4c, right). Similarly, the simulated image of the amorphous solid (Figure 4c, left) is in good agreement with the experimental image (Figure 4a, second frame), further confirming that HRTEM imaging can clearly distinguish between the two states.

The bonding energy of Pt with monovacancy and divacancy defects is very high, 7.26 and 7.21 eV, respectively.^30^ Therefore, in the temperature range of 20 to 800 °C that was investigated, the Pt atoms bonded to the vacancy defects cannot acquire enough kinetic energy for their displacement from either the thermal motion of the liquid or the direct momentum transfer from the electron beam (the maximum energy that can be transferred from the 80 keV electron beam to the Pt atoms is 0.97 eV, eq 1). As a result, the Pt atoms associated with the defects are firmly bonded to the graphene, which keeps them stationary during the time it takes to capture images, allowing them to display sharp atomic contrast in HRTEM images (Figure S12).

Our observations indicate that virtually every liquid nanoparticle contains some stationary Pt atoms bonded to defects in graphene, with others remaining free to move within the liquid. According to theoretical predictions, the crystallization of liquid nanodroplets is expected to have a significant activation barrier, leading to a melting-solidification hysteresis (Figure 3a, inset)^4,32^ with nucleation starting from the center, or the edges and propagating through the entire volume of the nanodroplet.^3,4,32^ We demonstrate that in our experiments, liquid Pt exists in an equilibrium with a crystalline nucleus, when a small number of stationary atoms are unable to disrupt the process, as shown by a time-series HRTEM imaging of platinum at 500 °C (Figure 3d). A crystal lattice consisting of 10–12 atomic planes forming directly from the liquid (such as in frames 4 and 13 s in Figure 3d) is metastable and undergoes melting and regrows several times before the nanoparticle solidifies fully (frame 26 s).

Using high electron beam flux irradiation, we can increase the number of defects in graphene around a specific liquid nanoparticle, enabling us to enhance the number of stationary atoms on-demand and investigate how they affect the dynamics of liquid metal solidification. The most unexpected phenomena arise in liquid Pt nanodroplets when a ring of stationary Pt atoms surrounds them. The liquid core corralled with stationary atoms remains liquid down to 200–350 °C, indicating a high activation barrier for crystal nucleation in this unusual state of metal. For example, crystallization from the edge is inhibited due to the constrained Pt–Pt distances dictated by the graphene’s dangling bonds, which do not match with any fcc crystal spacing. Consequently, the liquid-to-crystal nucleation pathways observed in free liquids are blocked in the corralled state, leading to experimental observations of a frustrated nucleation process, expressed by fluctuations between amorphous and liquid states of platinum (Figure 3e and Figure S10).

Additionally, there is a thermodynamic factor restricting the crystallization of corralled nanoparticles, stemming from the higher density of solid Pt than liquid, hence a negative volume change (Δ_*c*_ *V* < 0; Figure 4d) and exothermicity of crystallization (Δ_*c*_ *H* < 0) leading to negative pressure at the solidification point, according to the Clapeyron equation (d*p*/d*T* > 0, eq 4, Figure S13):

$$\frac{dp}{dT}=\frac{\Delta_{c}H\left(d\right)}{T\Delta_{c}V}$$ 4

Unlike free molten nanoparticles, the volume of corralled liquid cannot be easily adjusted. It requires either breaking the Pt–C bonds in the corral or straining the graphene sheet that has an in-plane Young’s modulus of 1 TPa^33^ - both extremely unfavorable. This provides an additional barrier for crystallization (Figure 4f).

We conducted molecular dynamics (MD) simulations to investigate the temporal evolution of a corralled liquid ca. 3 nm Pt nanoparticle at a constant temperature (Video S1), and its solidification dynamics as the temperature decreases (Videos S2, S3, and S4). For comparison, similar MD simulations were performed to ascertain differences in temporal atomic dynamics at a constant temperature of an uncorralled liquid Pt nanoparticle resting on graphene (Video S5) and its solidification dynamics as the temperature decreases (Videos S6 and S7). Our results indicated that while Pt atoms in the center of the corralled nanodroplet experienced significant displacements (red atoms, Figure 4e), those at the corral’s edges remained localized due to strong bonding with carbon atoms (blue atoms, Figure 4e). We simulated HRTEM images from the MD trajectories, matching well with experimental observations. The mobile Pt atoms in the center are not visible in HRTEM, revealing the graphene lattice, while stationary atoms at the edge show atomically sharp contrast (Figure 4e, leftmost image). HRTEM images simulated directly from the MD models correlated well with experimental data for a corralled liquid with some vacancy defects in the basal graphene layer (Figure 4g). As the temperature fell, the number of stationary atoms increased, leading to the formation of an amorphous solid nanoparticle (Figure 4g, last frame; Video S8), consistent with our C_c_/C_s_-corrected HRTEM experiments (Figure 4a, second frame). These results demonstrate that HRTEM imaging can effectively distinguish between the liquid and amorphous solid states of metal nanoparticles, thereby enabling the transition to be observed with atomic precision as a function of temperature.

For comparison, our MD modeling of the solidification dynamics of an uncorralled liquid platinum nanoparticle demonstrated a normal crystallization process, with a liquid-to-crystal transition (Figure S14 and Videos S6 and S7). The liquid metal forms a spheroidal shape to minimize surface tension, followed by the ordering of metal atoms into planes of an fcc lattice. To highlight the differences in atomic dynamics between corralled and uncorralled liquid metals, we analyzed the temporal evolution of these liquid particles at a constant temperature (Figures 4h, i). The unconfined liquid particle resting on graphene quickly relaxes into a spheroidal shape where most platinum atoms exhibit similar magnitudes of motion (Figure 4i). In the corralled state, on the same time scale, the liquid nanoparticle remains pinned to the edges, so that the motion of the atoms differs distinctly due to stationary platinum atoms around the edges (Figure 4h).

Upon cooling to around 200 °C, Pt–Pt bonding energy in supercooled liquid nanodroplets surpasses their kinetic energy, resulting in solidification of the corralled liquid metal into an amorphous state. Amorphous metal nanoparticles are known to be significantly less stable than crystalline ones,^2,4^ but in our experiments, their crystallization can occur only when the corral breaks, either spontaneously at 200 °C (Figure 4a, last frame) or by electron beam stimulation at 30 °C (Figure S10, last frame). Larger corralled liquid particles of ca. 20 nm in diameter, however, can partially crystallize without breaking the corral, with a crystal phase forming at the center, while the edges remain amorphous (Figure 4b). Distributing the structural tension of the amorphous solid phase across a larger number Pt atoms appears to have a stabilizing effect in nanoparticles.

While this study focuses on platinum on carbon due to its technological significance, our methodology has also been extended to palladium and gold nanoparticles on graphene, prepared by the same method, which have a size distribution of 2–5 nm (Figure S5). Similar to platinum, both Pd and Au are liquid at 750 °C, losing the atomic definition of the metal lattice, with some metal single atoms attached to vacancy defects (leftmost frames in Figure 5 and Figures S5 and S6). However, unlike platinum, Au has a lower energy of bonding to the graphitic carbon (2.97 eV for Au vs 7.41 eV for Pt), which limits the ability to control the number of vacancy defects and, consequently, the number of stationary Au atoms. Accordingly, even a high flux of 80 keV electron beam cannot create a sufficient fraction of stationary atoms to alter the solidification pathway of gold (Figure 5a).

**Figure 5:**
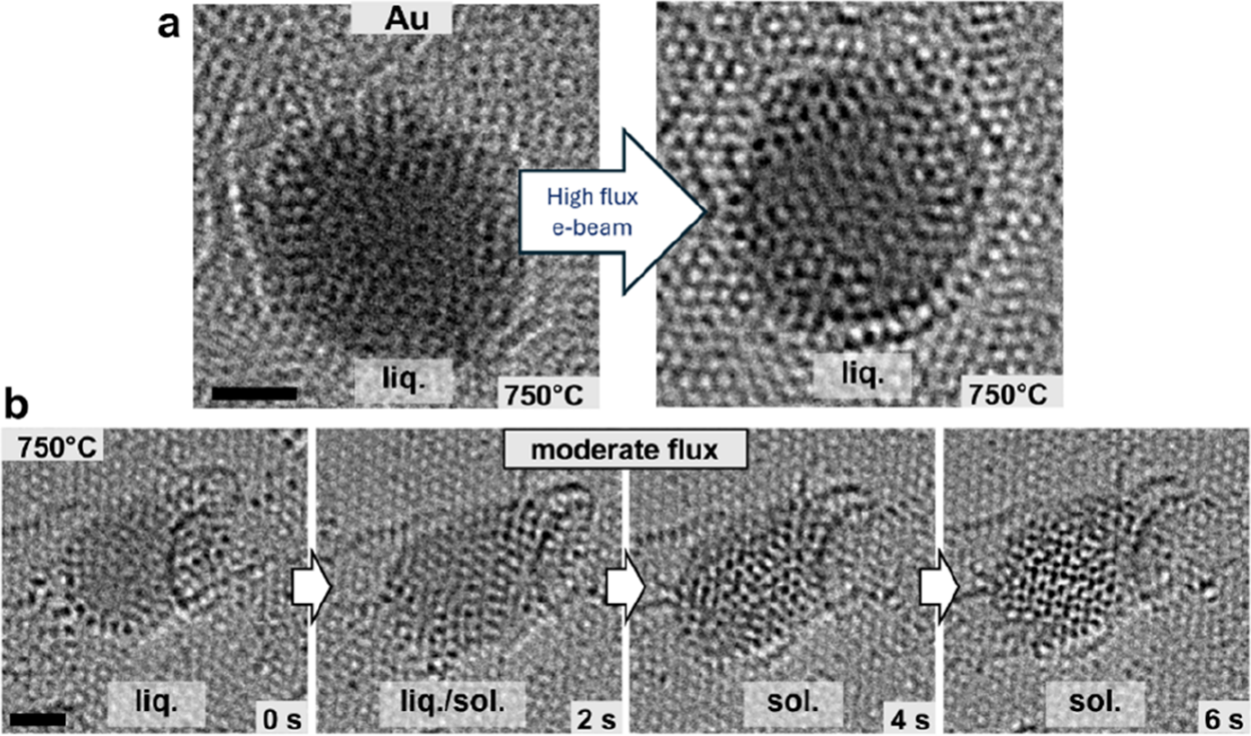
Behavior of liquid Au and Pd under electron beam. Examples of HRTEM images of liquid Au and Pd nanoparticles at 750 °C. (a) A liquid nanoparticle of gold undergoes minimal changes under the moderate or high flux of the electron beam (right image after high flux irradiation), remaining largely liquid with no distinct phase transformations. (b) In contrast, a liquid nanoparticle of Pd undergoes very fast transformations already under the moderate flux of the electron beam, making it difficult to study the effect of the stationary atoms on the solidification process. (scale bars: 1 nm).

If we compare Pd to Pt, while the valence electron shell remains same, ensuring the substantial energy of palladium–carbon bonding (5.46 eV)^30^ which allows defect formation rates similar to Pt, palladium’s lower atomic mass means that it received almost twice as much kinetic energy as platinum from the electron beam (1.78 eV for Pd vs 0.97 eV for Pt, eq 1). The increased kinetic energy passed from the incident electron to palladium allows the atoms to be more mobile. Therefore, the crystallization process cannot be sufficiently interrupted by the presence of stationary Pd atoms to keep its rate commensurate with the HRTEM image capture rate. Our measurements indicate that even a moderate electron beam flux induces crystallization in Pd nanoparticles, without changing the temperature, making the onset of this process unpredictable (Figure 5b). Overall, among the three transition metals studied, platinum phase transitions exhibited the most suitable atomic dynamics for real-time image capture using HRTEM.

## Conclusions

We investigated atomic-scale dynamics during phase transitions in liquid metals. Spherical and chromatic aberration-corrected HRTEM imaging can differentiate between molten and solid nanoparticles, and it can detect, through the liquid phase, that some individual metal atoms are trapped at defect sites on the graphene support. These stationary metal atoms, which remain bonded to the support, are present in all liquid nanoparticles across the studied temperature range with varying proportions, depending on the local environment of the nanoparticle. We demonstrated that a higher concentration of vacancy defects in graphene—conveniently created by a condensed, high-flux 80 keV electron beam—around a liquid nanoparticle increases the number of stationary atoms. These stationary atoms were discovered to significantly influence the solidification processes, leading to the formation of metastable crystal nuclei and the coexistence of solid amorphous and crystalline phases within nanoparticles, all occurring in the same area of the sample under the same experimental conditions.

The diverse behaviors of nanoparticles on carbon raise many questions about our current understanding of the atomistic mechanisms that govern adsorption or catalysis by metal nanoparticles at elevated temperatures. Most existing concepts, based on ensemble averaging analysis, may be too simplistic to accurately represent the diversity of atomic dynamics we observed. Notably, as the number of stationary atoms increases, they can form a corral around the liquid, resulting in unusual phenomena such as a supercooled liquid metal state, which can exist 1000 °C below the normal solidification temperature. Previously, corralling at the nanoscale was possible only for electrons^34^ and photons,^35^ resulting in significantly altered behavior. In our study, we created a liquid in an atomic corral that revealed unusual phase behavior of metal. This effect was particularly expressed in platinum on graphene, which could be harnessed in designing the next generation of self-regenerating catalysts or energy materials based on Pt on carbon supports in the future.

## Methods

### Nanocluster Formation on Graphene

For platinum deposition, a graphene grid or heated SLG chip was placed on a stainless steel holder and exposed to atomically dispersed metal from magnetron sputtering at room temperature. AJA magnetron sputtering was used at 10 mTorr (Ar gas), 35 W for Pt, with a 2s deposition time. CVD graphene on copper substrate from Graphenea was etched with a 6% ammonium peroxodisulfate solution, and the SLG was transferred to a TEM grid or chip by fishing it from the solution’s surface. The successful sample preparation was confirmed by energy dispersive X-ray (EDX) analysis (Figure S15).

### Aberration-Corrected High-Resolution Transmission Electron Microscopy

HRTEM images were acquired with an image-side *C*_c_/*C*_s_-corrected SALVE microscope operated at 60 or 80 kV with resolutions of 90 or 76 pm, respectively (SALVE: subangstrom low-voltage electron microscopy). The SALVE *C*_c_/*C*_s_ corrector adopts a quadrupole–octupole design, which corrects the geometrical axial aberrations up to the fifth order, off-axial aberrations up to the third order, and chromatic aberration.^36^ Data acquisition was conducted with a Ceta CMOS camera.

### Electron Beam Flux Estimation

Since the high-dose conditions are too extreme to image directly on the TEM camera, the flux was estimated from the viewscreen, through the screen current, and the number of electrons passing through the illuminated area. Accurate information on the intensity distribution over the illuminated area cannot be obtained from the viewscreen, but the illuminated area can be estimated (Figure S16). With a 15 nA (9.36 × 10^10^ e/s) screen current and an illuminated area of 12 nm radius, the resulting flux equates to 2.07 × 10^8^ e/(nm^2^ s). For 60 kV the maximum flux (high flux) is estimated to be on the order of ∼ 6 × 10^7^ e/(nm^2^·s). For 80 kV a higher flux can be achieved (2 × 10^8^ - 1 × 10^9^ e/(nm^2^·s). The moderate flux that was used for imaging in both cases is ∼ 10^6^ e/(nm^2^·s).

### Heating Regime

For in situ heating experiments, we utilized FEI’s NanoExi/v heating holder, equipped with eight electrical contacts for simultaneous heating/biasing. The MEMS chips feature 22 windows with 5 μm diameters, each containing a 15 nm thick amorphous holey silicon nitride film. A CVD graphene monolayer was deposited on the chips, allowing the observation of Pt atoms and nanoparticles within the silicon nitride film’s holes. The system offers a temperature accuracy of 4% and can exceed 1200 °C. We employed a low cooling rate of 0.5 °C/s to minimize the sample drift that can displace observed particles out of the field of view.

### Computational Methods

The dynamics of corralled liquid Pt nanoparticles were simulated using analytical potential molecular dynamics (MD), as implemented in the LAMMPS package.^37^ A supercell containing a bilayer graphene (6856 carbon atoms), with a few-nm hole in the top layer was filled with molten Pt (430 Pt atoms). The outermost carbon atoms at the supercell boundaries were kept fixed throughout the simulations. The Pt–Pt interactions were defined with the Embedded Atom Method (EAM)^38^ to achieve accurate modeling of the melting/crystallization, and the Pt–C interactions were described with the Modified EAM (MEAM).^39^ Despite the accuracy of the EAM potential in modeling the dynamics of Pt-only systems, combining it with the MEAM potential to include Pt–C interactions introduced deviations from the experiment, meaning that the experimental Pt solid–liquid behaviors were possible to theoretically capture at higher temperatures. Elevating the temperature, however, caused minor instabilities in the terminated carbon atoms at the edges of the corral, providing them with enough energy to be detached, either to be sputtered or mixed with the Pt nanoparticle. To address this issue, we allowed the carbon atoms to move freely only in the vertical (z) direction, and the occasionally detached carbon atoms were allowed to evaporate out of the supercell. This approximation not only helped to tackle the carbon-detachment issue but also amplified the confining role of the corral on phase transitions in liquid metal, as observed in experimental data. This allowed us to evaluate our hypothesis more effectively and to reduce the time required to observe the Pt solid–liquid transition to occur within a few nanoseconds.

The simulations of the Pt atoms, in the corralled nanoparticle case, were performed under the canonical ensemble (NVT), while the carbon atoms were treated with free MD (i.e., plain time integration to update positions and velocities). The MD time step was set to 1.0 fs, and temperature variations were introduced in steps of 100 degrees, with 0.5 ns equilibration time in each step.

### TEM Image Simulations

HRTEM image simulations were performed with abTEM,^40^ which uses a multislice algorithm. Atomic models for the image simulations are created using the Atomic Simulation Environment (ASE).^41^ Simulation parameters are comparable to the typical experimental conditions (i.e., pixel size, defocus, aberrations, etc. - example parameters in Table S1). For creating simulated TEM images of liquid Pt nanoparticles, first, the model of an amorphous Pt nanoparticle was created. For this, Pt atoms were placed at random positions within a given volume (for instance, a cylinder with a 3 nm diameter). To ensure a minimum distance between the atoms, a new atom was only placed if there was no already placed atom closer than a set minimum distance (here 0.2 nm). Then repeat the following steps: (a) remove the amorphous nanoparticle; (b) create a new nanoparticle within the same volume as the previous one; (c) simulate a TEM image from the amorphous nanoparticle. After a sufficient number of simulated images were generated (here, 250), the images are summed up, thus mimicking the nature of a liquid Pt nanoparticle and demonstrating the electron beam transparency of liquid metal, as observed in experimental HRTEM images.

## Supplementary Material



















## Data Availability

All data are available in the main text or the Supporting Information.

## Notes

Figures S1 to S16 include the effect of Pt on vacancy defect formation in graphene, additional HRTEM images of Pt, Pd and Au particles at different temperatures and electron beam fluxes, thermodynamics of phase transition in corralled nanodroplets, theoretical modeling of the solidification dynamics of unconfined Pt on defective graphene, and EDX spectrum. Table S1 includes TEM image simulation parameters (PDF)
Video S1_temporal evolution of corralled liquid Pt_top view (MP4)
Video S2_solidification dynamics of corralled liquid Pt_side view (MP4)
Video S3_solidification dynamics of corralled liquid Pt_perspective view (MP4)
Video S4_solidification dynamics of corralled liquid Pt_top view (MP4)
Video S5_temporal evolution of unconfined liquid Pt_top view (MP4)
Video S6_solidification dynamics of unconfined liquid Pt_side view (MP4)
Video S7_solidification dynamics of unconfined liquid Pt_top view (MP4)
Video S8_simulated HRTEM of solidification dynamics of corralled liquid Pt (MP4)

## §

German Reference Office for Proficiency Testing and Reference Materials (DRRR GmbH), Reinhartser Str. 31, 87437 Kempten (Allgäu), Germany
