# Supplementary material for: Stationary Atoms in Liquid Metals and Their Role in Solidification Mechanisms
Source: ACS Nano. 2025 Dec 9;19(50):42002–12. doi: 10.1021/acsnano.5c08201 (PMC12752686; doi:10.1021/acsnano.5c08201)
Supplement: Supplementary file 1 [file nn5c08201_si_001.pdf]

Supplementary Materials for

Stationary Atoms in Liquid Metals and Their Role in Solidification Mechanisms

Christopher Leist,[1,2] Sadegh Ghaderzadeh,[3] Emerson C. Kohlrausch,[3] Johannes Biskupek,[1] Luke T. Norman,[3] Ilya Popov,[3] Jesum Alves Fernandes,[3] Ute Kaiser,[1,4]* Elena Besley,[3]* Andrei N. Khlobystov[3]*

[1]Electron Microscopy of Materials Science, Central Facility for Electron Microscopy, Ulm University; Ulm 89081, Germany
[2]German Reference Office for Proficiency Testing and Reference Materials (DRRR GmbH), Reinhartser Str. 31, 87437 Kempten (Allgäu), Germany
[3]School of Chemistry, University of Nottingham; Nottingham, NG7 2RD, United Kingdom
[4]Institute for Quantum Optics (IQO), Ulm University; 89081 Ulm, Germany

Activation of carbon displacement in graphene by Pt atoms

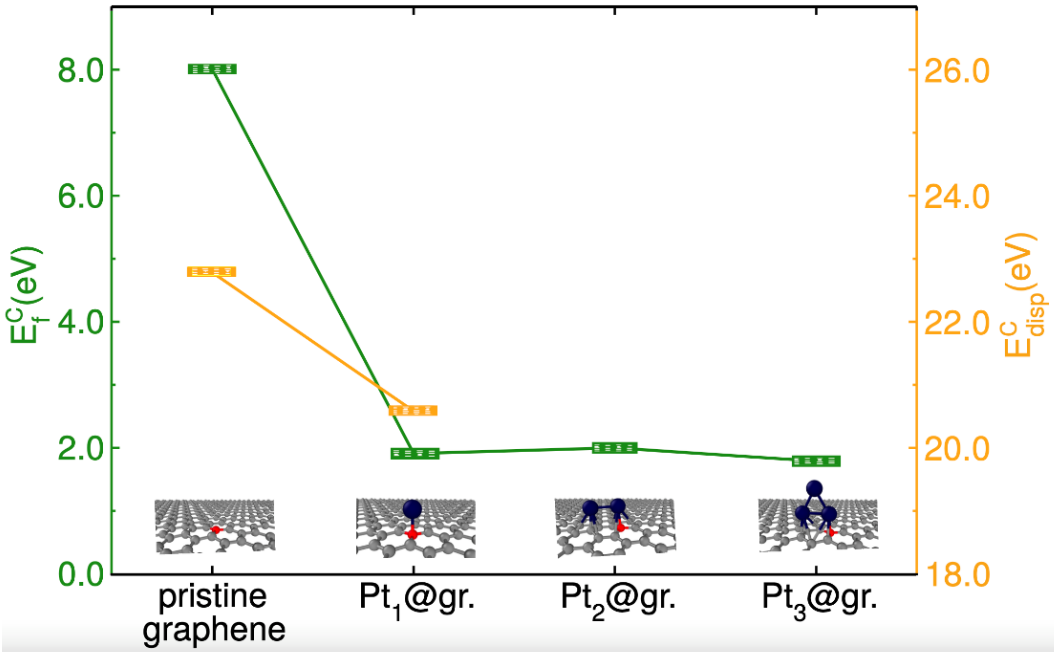

Figure S1. Pt atoms bind strongly to the dangling bonds in graphene, both decreasing the formation energy of vacancy defects ($E_f{}^C$) and the barrier for carbon atom displacement ($E_{disp}{}^C$), calculated by DFT/ab-initio MD (green and orange plots are formation energies and energy thresholds for carbon atom displacement in graphene in the absence and presence of Pt respectively).

Spin-polarized Density Functional Theory (DFT) calculations of carbon-vacancy formation energies in graphene were performed with the Vienna Ab initio Simulation Package (VASP) (*1*, *2*), within the plane-wave projector augmented-wave (PAW) method. The structures were relaxed using the Perdew–Burke–Ernzerhof (PBE) exchange–correlation functional (*3*) with a force tolerance of 0.005 eV$Å^{-1}$ and an electronic convergence criterion of 10^{-6} eV. The energy cut-off was set to 550 eV, and a gamma-point-centred Monkhorst-Pack k-point grid of 12×12×1 was used to sample the Brillouin zone. Van der Waals interactions were considered using the DFT-D3 method (*4*) with Becke–Jonson damping function. The pristine graphene supercell contained 98 carbon atoms.

The vacancy formation energies were calculated as follows (*5*):

$$E_f(C) = (E_{V_C+Pt} - E_{graphene+Pt} + \frac{E_{graphene}}{n}),$$

where E_{V_C+Pt} is the total energy of the defective system (i.e., Pt atom on the graphene with vacancy),
$E_{graphene+Pt}$ is the total energy of a Pt atom on a pristine graphene sheet, and $\frac{E_{graphene}}{n}$ is the normalized (per number of carbon atoms n) total energy of the pristine graphene system.

The threshold displacement energies of carbon atoms in the absence/presence of Pt atoms were calculated using ab initio molecular dynamics (AIMD), with the CP2K package (*6*), based on PBE functional and a hybrid Gaussian/Plane-Wave scheme (GPW) (*7*). The valence electrons were expanded in Double-Gaussian basis sets with one polarization function (DZVP) optimized for multi-grid integration (*8*), while the core electrons and nuclei were described by Goedecker-Teter-Hutter (GTH) pseudopotentials (*9*). Four multi-grids and a cutoff of 300 Ry were used in this study. The simulation cell contained 160 carbon atoms in the pristine graphene system. The geometry of all the structures was carefully optimized.

Experimental evidence for Pt-promoted defect formation in a single-layer graphene is obtained from time-series AC-HRTEM imaging of a naked Pt particle positioned on the plane of graphene which is irradiated with a high-intensity 80 keV electron beam. Carbon atoms are displaced in the vicinity of Pt nanoparticle and the size of the defect increases proportionally to the total dose of the electron beam. Pt atoms bind to the open edge of the defect due to strong covalent Pt-C bonding.

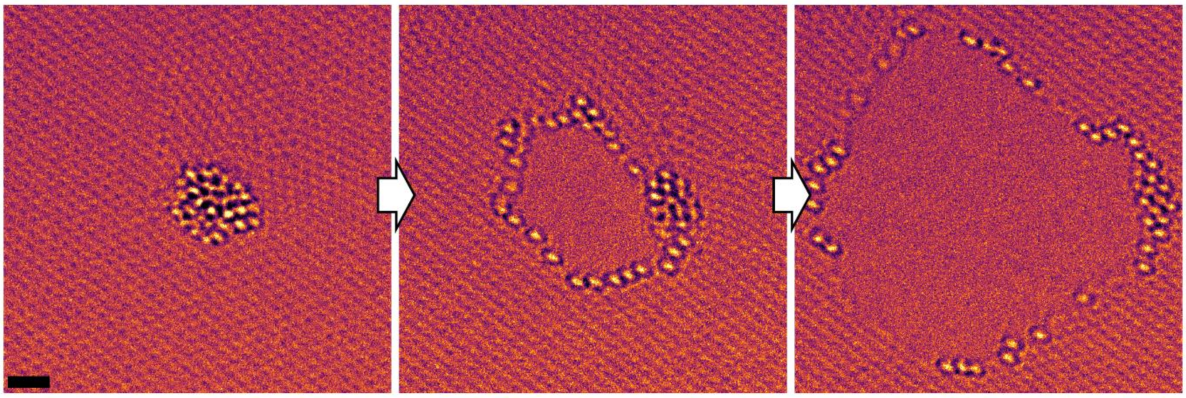

Figure S2. Cc/Cs-corrected HRTEM image time series showing the formation and propagation of a vacancy defect in graphene caused by irradiation with a high flux 80 keV electron beam at 20 °C in between the frames: as the defect grows, Pt atoms bind to the open edges (bright atoms). (scale bar: 1nm; false colour is applied for clarity)

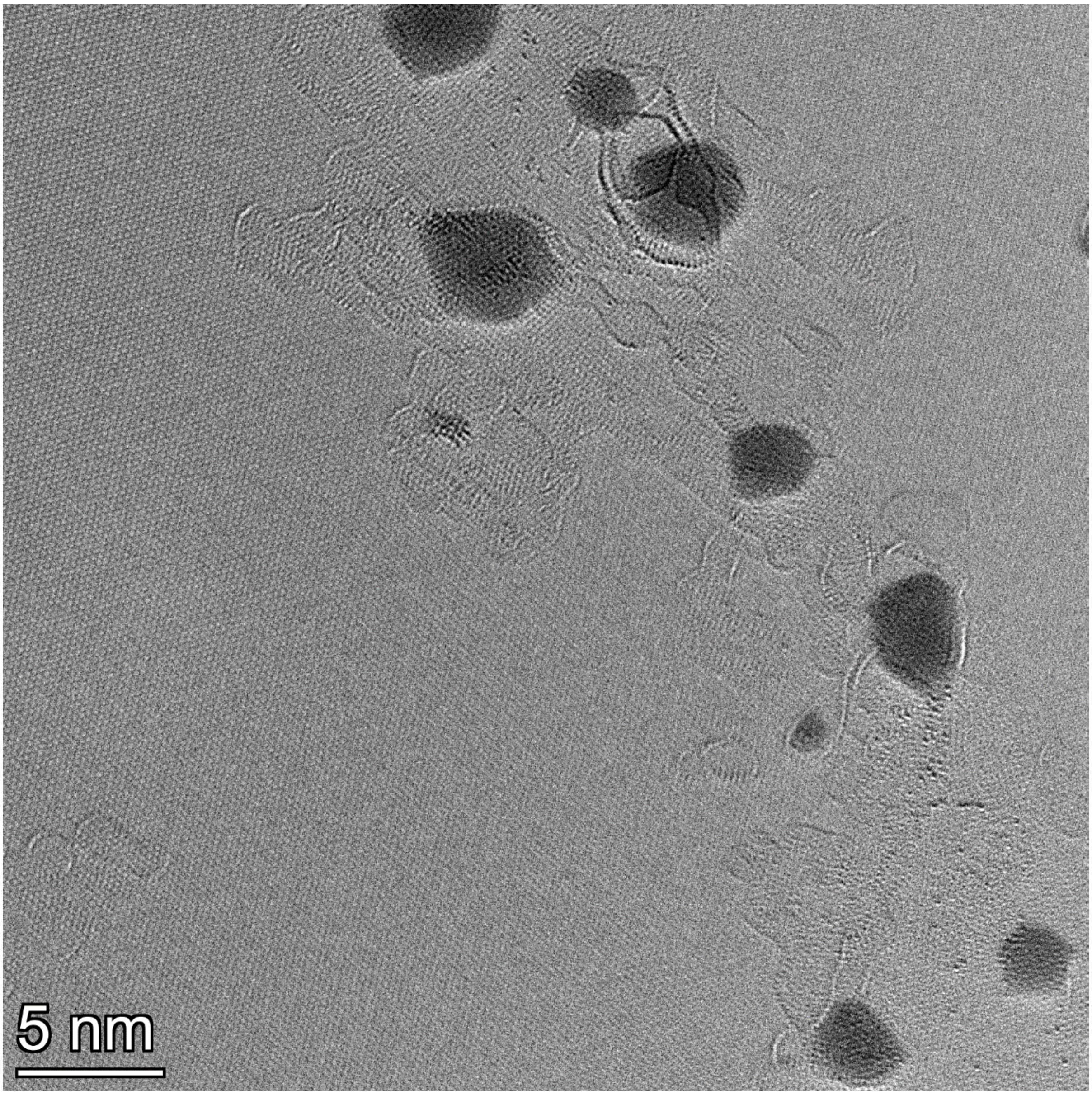

Figure S3. 80kV Cc/Cs-corrected-HRTEM image taken at 900°C. No high flux has been applied, and all Pt particles are liquid.

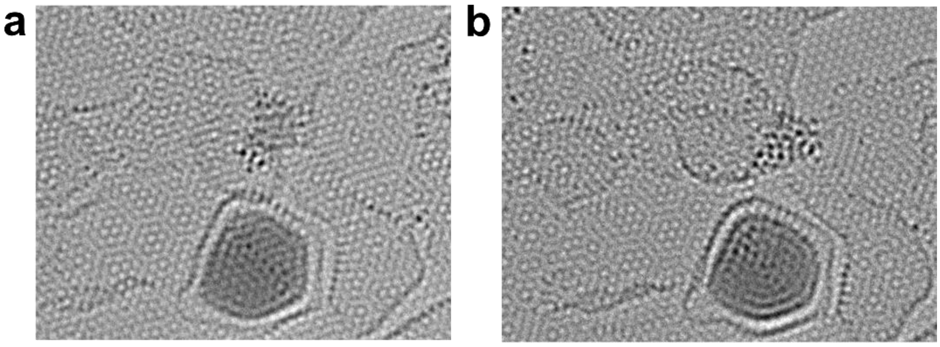

Figure S4. Protective Carbon shell prevents solidification by 80kV e-beam. (a)750°C 80kV Cc/Cs-corrected HRTEM image showing two Pt clusters one of which is encapsulated by carbon, both are liquid. (b) 750°C 80kV Cc/Cs-corrected HRTEM image of the same clusters after exposure to high flux e-beam. The encapsulated Pt particle remains liquid, and the unencapsulated particle solidifies.

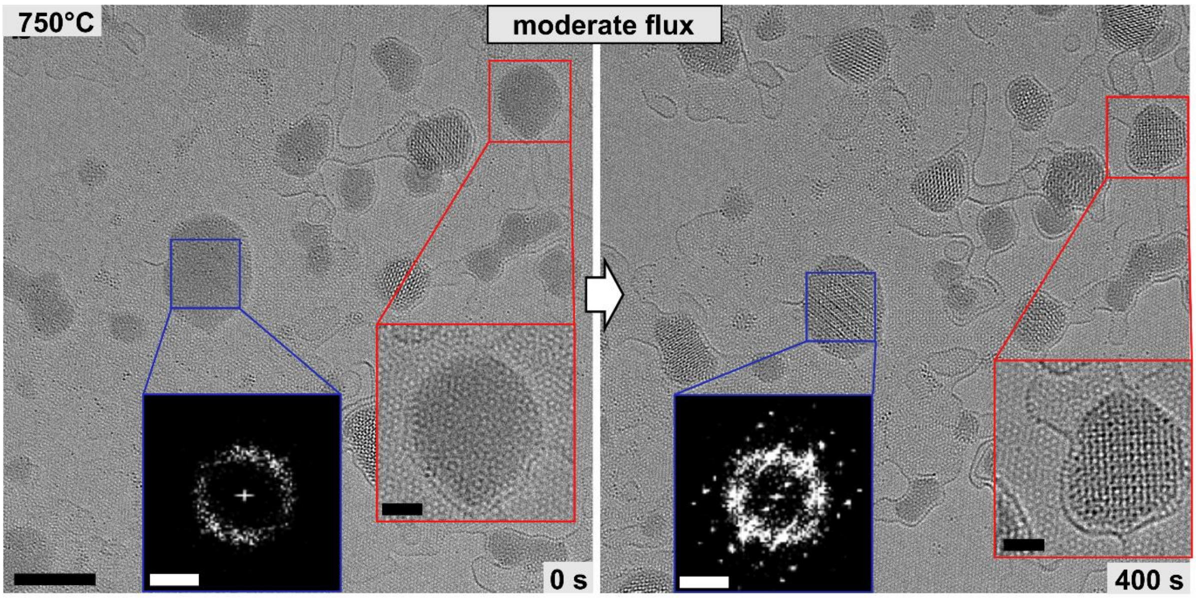

Figure S5. 80kV Cc/Cs-corrected-HRTEM image taken at 750°C showing multiple Pd-nanoclusters some already crystalline and some liquid. When exposed to moderate flux for imaging liquid nanoclusters turn crystalline substantially increasing their number (scale bar: 5nm). Red boxes: Zoom in of a cluster turned crystalline during the experiment (scale bars: 1nm). Blue boxes: FFTs of a cluster turning crystalline (scale bars: 5 nm^{-1}).

.

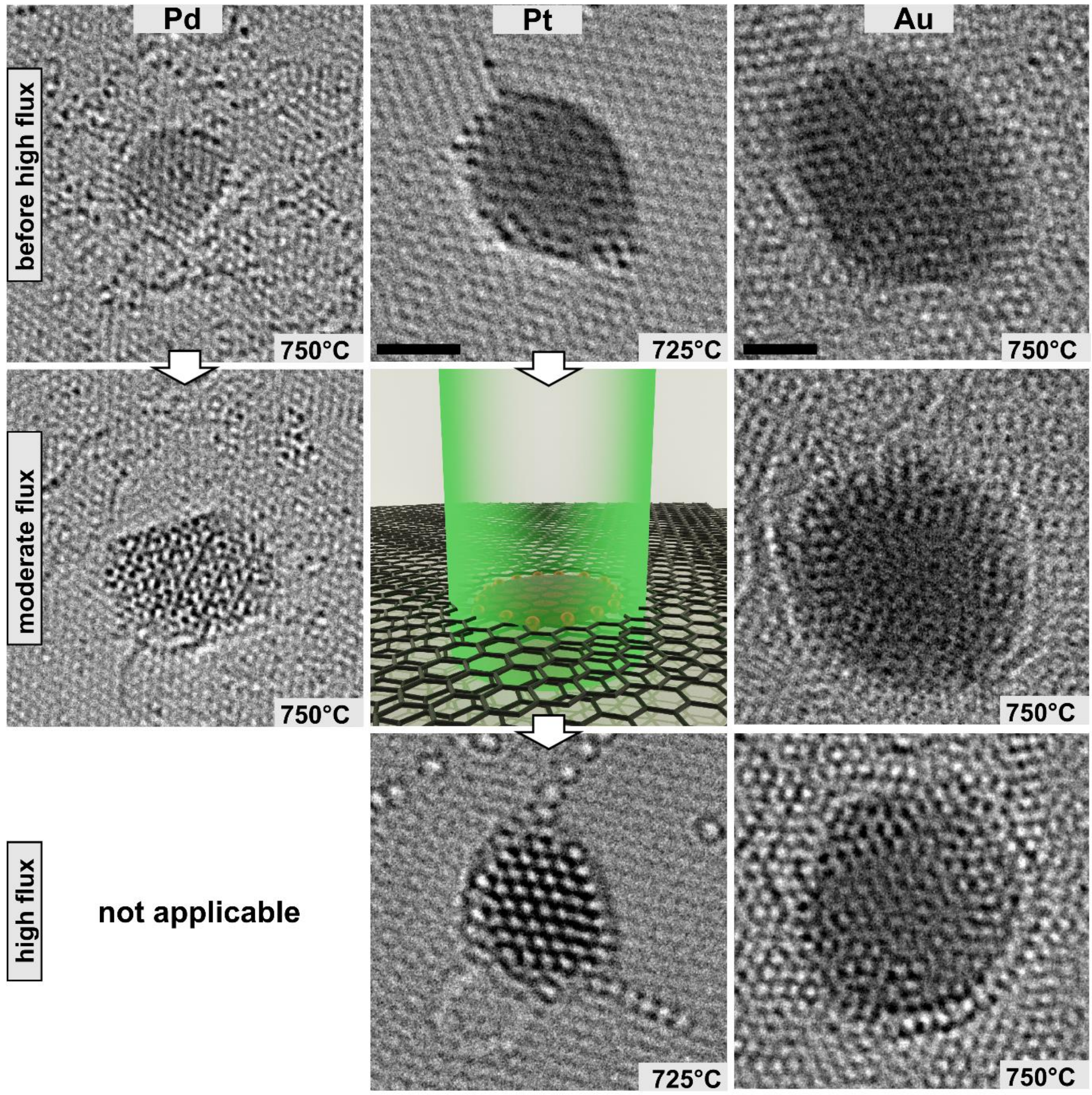

Figure S6. 80kV Cc/Cs-corrected-HRTEM images showing the different behaviors of different molten metal nanoclusters under the electron beam. First row: all metal clusters are liquid at temperatures above 700°C. Second row: under moderate flux Pd crystalizes while both Pt and Au remain liquid. Third row: High flux allows Pt to crystalize while gold still remains liquid.

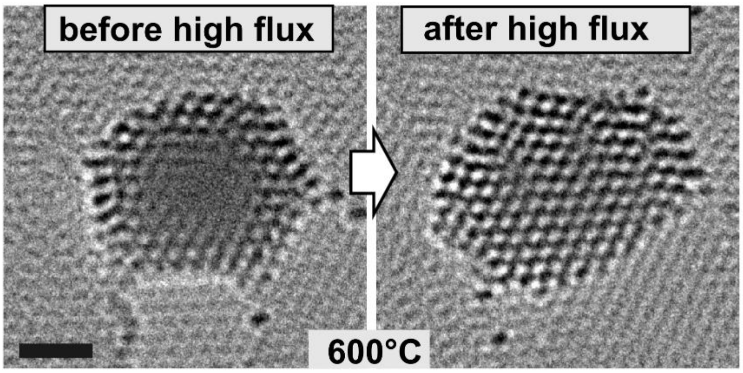

Figure S7. 80kV Cc/Cs-corrected-HRTEM images showing a Pt- corralled liquid before and after high-flux treatment at 600°C. The before and after images were recorded using medium electron fluxes to prevent detector damage. High-flux causes the corral to fully crystalize (scale bar: 1 nm).

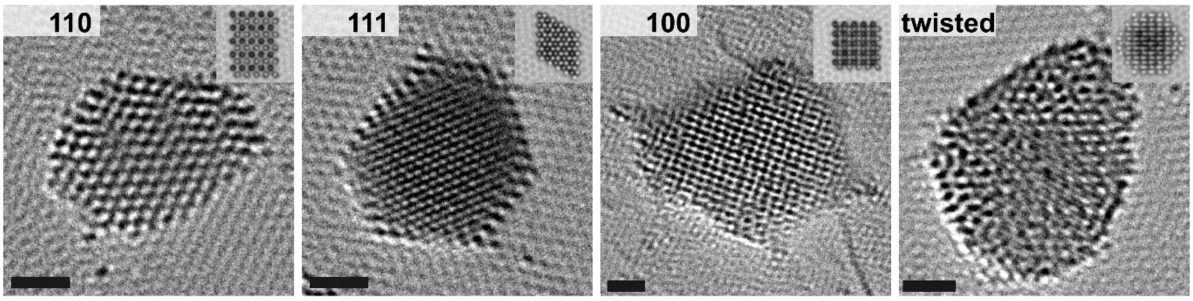

Figure S8. 80kV Cc/Cs-corrected-HRTEM images showing fcc lattice of Pt- nanoparticle with different orientations toward graphene as indicated. The pattern and lattice spacing fit with HRTEM image simulations aligned to the proposed direction. The respective image simulations of matching orientation in the top right corner (scale bar: 1nm)

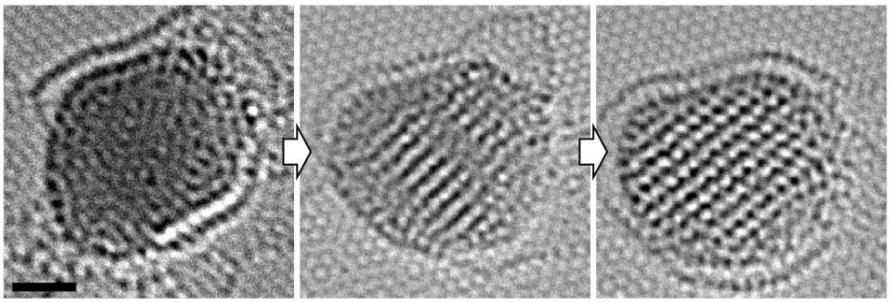

Figure S9. Time series of 80kV Cc/Cs-corrected-HRTEM images showing the crystallization of liquid nanoparticles with a small number of immobilized atoms at d 700 °C. They exhibit classical one-step nucleation of fcc Pt directly from the liquid phase, which exists in an equilibrium (crystal planes of the nucleus can re-melt and assemble again in a different direction).

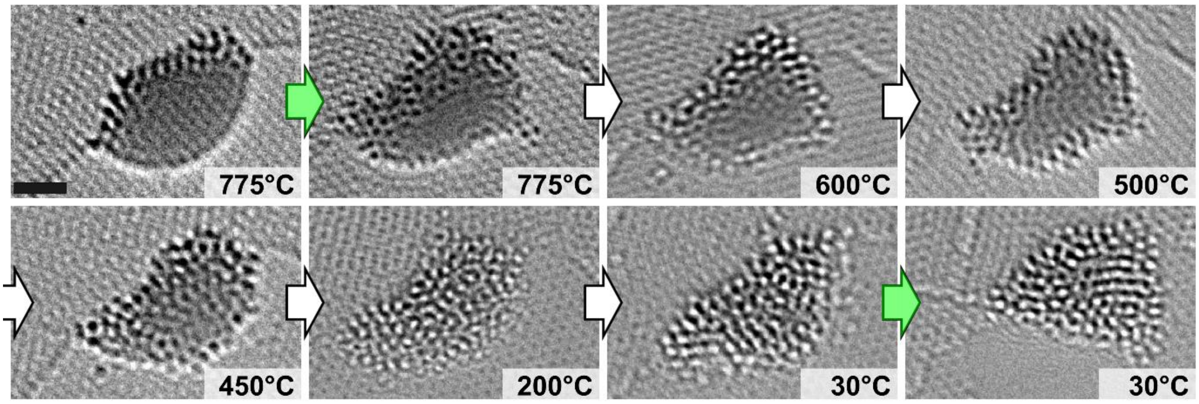

Figure S10. A series of 80kV Cc/Cs-corrected-HRTEM images showing the conversion of a liquid nanoparticle to a corralled state by 80 keV electron beam irradiation at 775°C, which subsequently solidifies to amorphous at 200°C, then transitions to an fcc crystalline state by additional electron beam irradiation at 30°C with the side effect of hole creation in the graphene. The green arrows indicate high flux treatment between the images (scale bar: 1nm).

Full frame HRTEM image series showing cooling of Pt-nanoparticles and the corralled Pt nanoparticle described in the main text

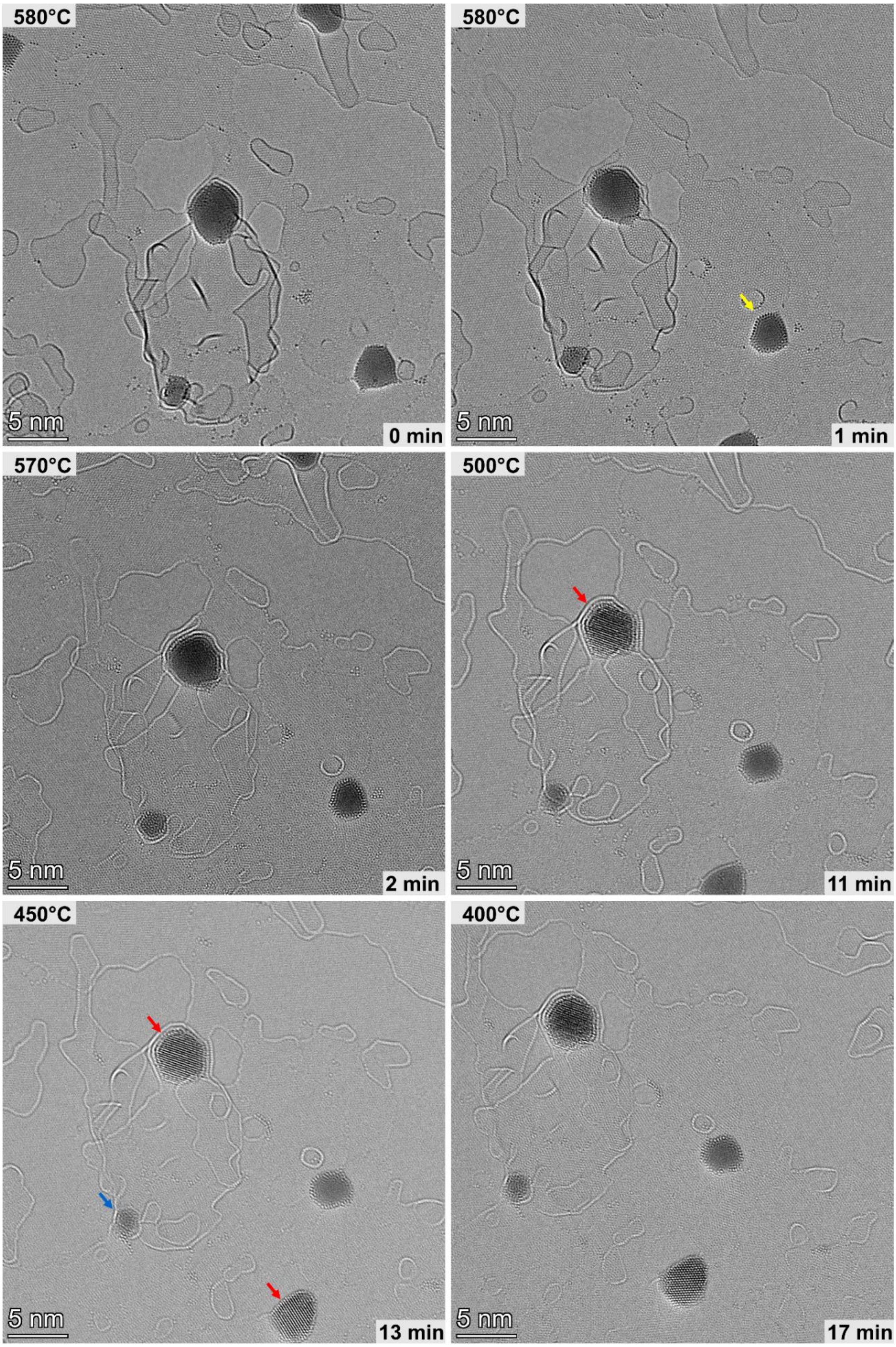

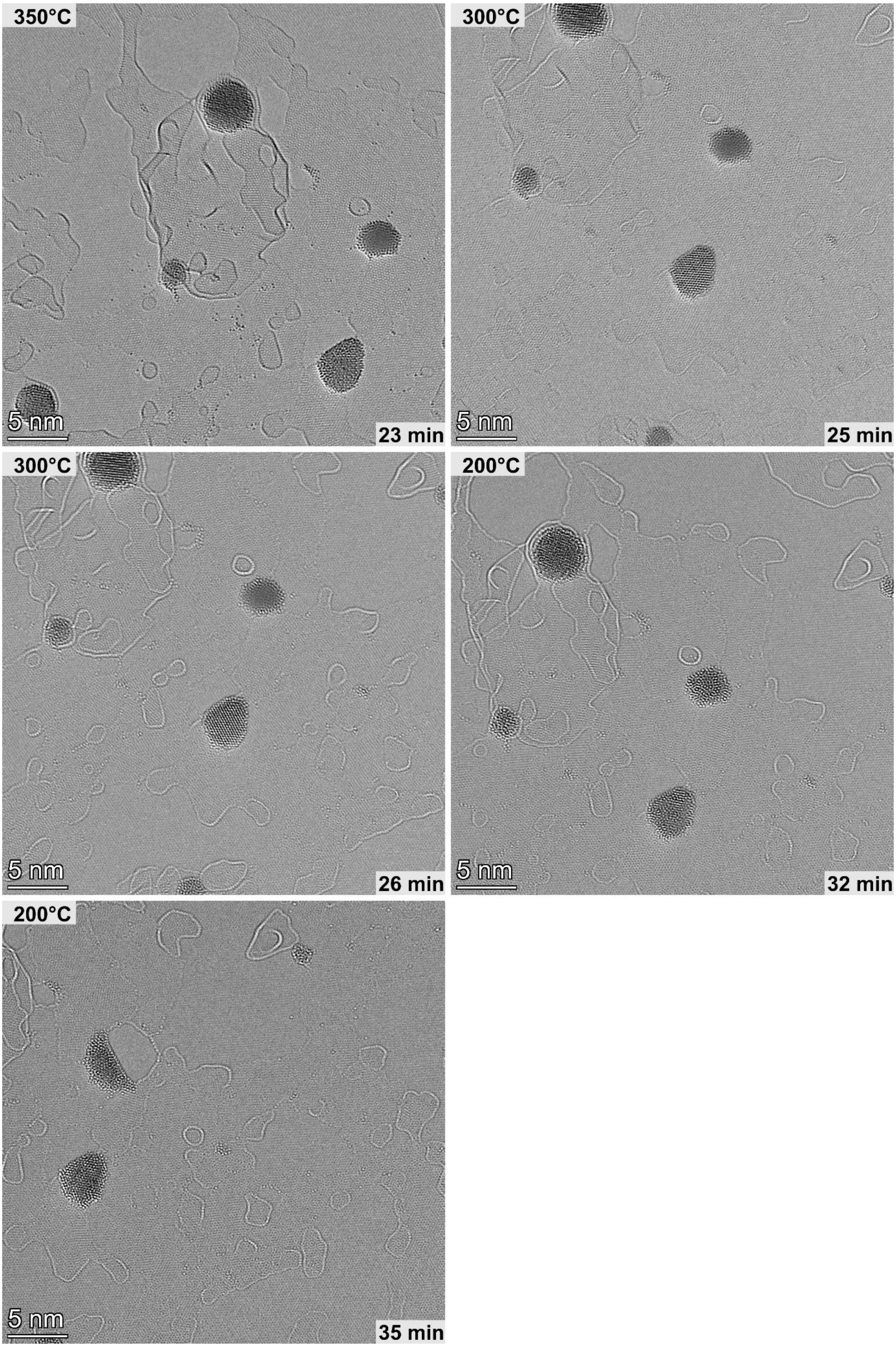
350°C
5 nm
23 min
300°C
5 nm
25 min
300°C
5 nm
26 min
200°C
5 nm
32 min
200°C
5 nm
35 min

Figure S11. 80kV Cc/Cs-corrected-HRTEM image series. During this series the sample is cooled from 580°C where all Pt nanoparticles are molten to 200°C solidifying the nano clusters in the process. Of special notice is a corralled liquid Pt nanoparticle (yellow arrow) created by high flux irradiation. The center of this particle solidified significantly later than all others. The sample was cooled in steps between the images with a cooling rate of 0.5°C/s.

Pannel 1: 580°C, all particles are liquid

Pannel 2: 580°C, high flux before the image acquisition causes a corral to form (yellow arrow)

Pannel 3: 570°C, cooling begins

Pannel 4: 500°C, the largest cluster is crystalline (red arrow)

Pannel 5: 450°C, all clusters except for the corral are solid. The clusters marked with red arrows are crystalline while the smallest cluster (blue arrow) solidifies into an amorphous cluster possibly due to the surrounding carbon shell.

Pannels 6-9: 400-300°C, the coral remains liquid while the other particles remain solid

Pannel 10: 200°C, corralled liquid nanodroplets solidifies into the amorphous phase as described in Figure 4 and the main text.

Pannel 11: 200°C, the amorphous cluster crystalizes tearing the graphene substrate in the process as described in Figure 4 and the main text.

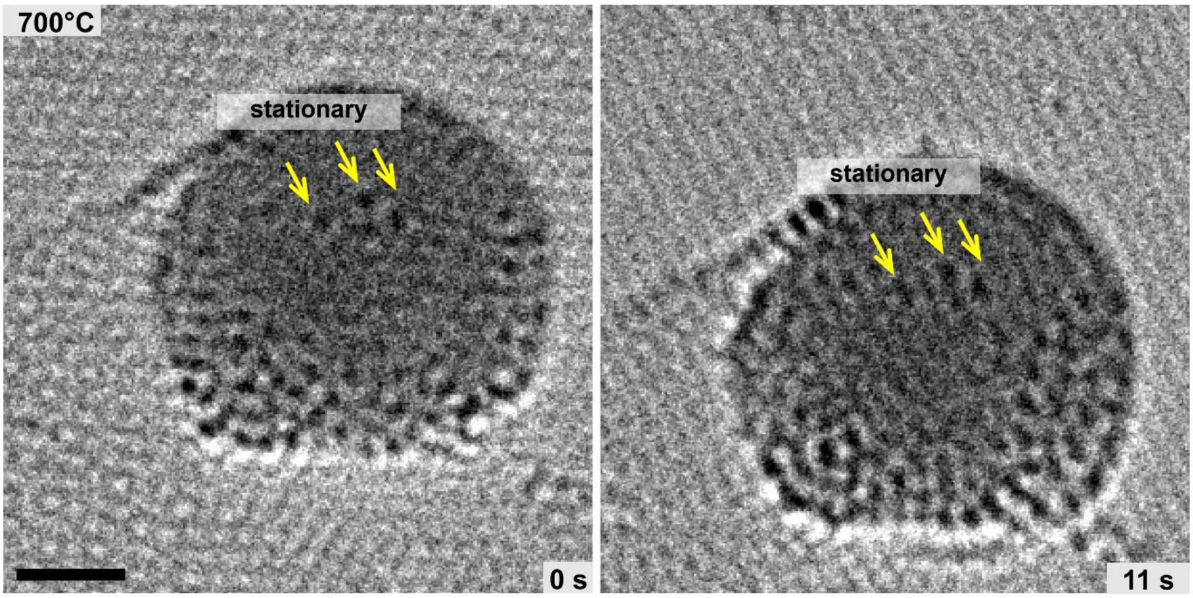

Figure S12. 80kV Cc/Cs-corrected-HRTEM showing stationary atoms inside a liquid Pt nanoparticle which do not change their position for several seconds.

Thermodynamics of phase transition of corralled nanodroplets

For the bulk phase of platinum, the coexistence line separating liquid and solid states in a pressure-temperature (p-T) phase diagram is described by the Clapeyron equation

,

where $\Delta_c H$ and $\Delta_c V$ are the changes in the molar enthalpy and molar volume in the crystallization process. Signs of these two quantities entering the right-hand side of the equation are negative: $\Delta_c H <$ 0 because crystallization is an exothermic process and $\Delta_c V < 0$ because the solid phase of Pt has a higher density than liquid (21.45 vs 19.77 g/cm^3), which means that the metal shrinks as it crystallizes. It means that the slope of the coexistence curve is positive (dp/dT > 0), and lower crystallization temperatures correspond to lower pressures. This agrees with the experimental phase diagram of Pt **(Figure S12)**. Although the solid-liquid coexistence temperature shifts down to c.a. 600-700 ^{o}C for the size of nanodroplets in our experiments because of the Gibbs-Thomson effect, we expect that the general conclusion about its incline remains valid in this case too. We use this result in the discussions of the main text.

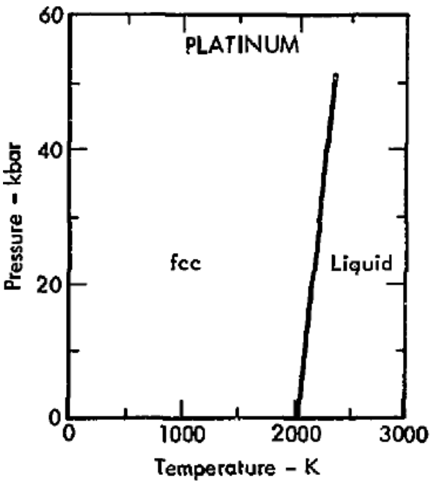

Figure S13. Platinum phase diagram, reproduced from "Phase Diagrams of the Elements", David A. Young, UCRL-51902 (copyright free).

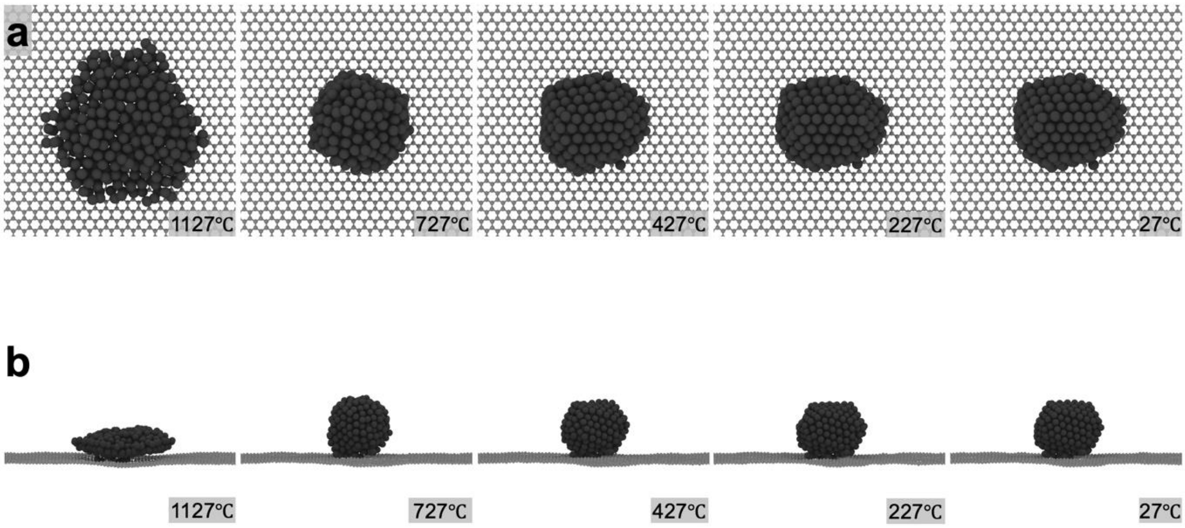

Figure S14. Solidification dynamics of unconfined Pt on defective graphene – (a) top view and (b) side view. The temperature varies from 1127℃ to 27℃. See full videos (top and side views) in Supplementary Video Files 6 and 7. The colours of Pt atoms indicate displacements from original positions: red – large displacement, blue – small displacement.

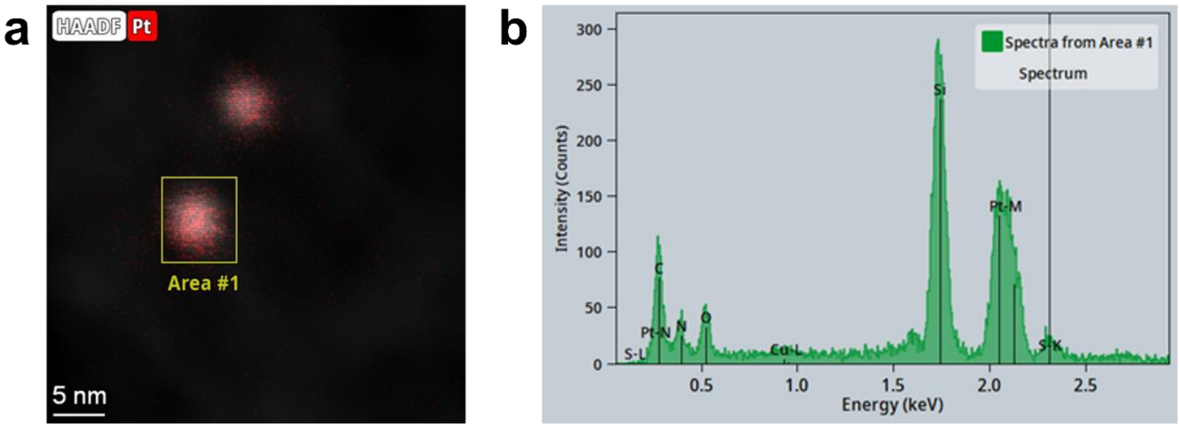

Figure S15. EDX analysis of Pt clusters on MEMS chip. a) EDX mapping of Pt clusters on a MEMS chip. b) Integrated EDX spectra of Area #1.

Scanning transmission electron microscopy (STEM) coupled with (EDX) analysis was carried out with TEM Thermo Fisher Talos 200X with a dedicated SuperX EDX detector, operated at an accelerating voltage of 80 kV. The Si and N signals in EDX arise from the MEMS chip's silicon nitride membrane.

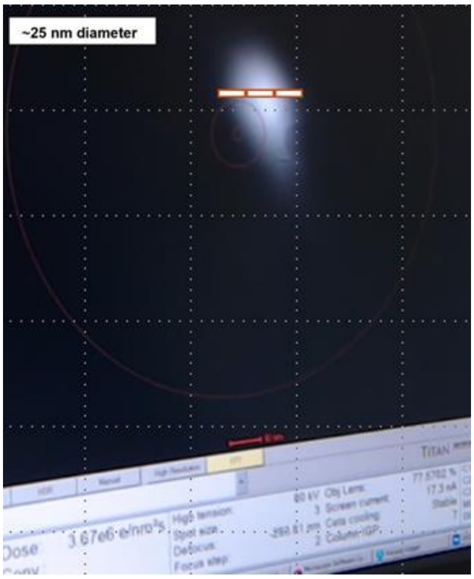

Figure S16. Image of the microscope's view screen on the control monitor during high flux treatment. Direct imaging on the camera during high flux is not possible due to potential equipment damage. The illuminated area is estimated by placing boxes of the same size as the red scalebar in the image's centre on the illuminated area.

Table S1. Example image simulation parameters

**Simulation parameters**	**Values**
sampling	0.09 Å/pix
acceleration voltage	80 kV
defocus	10 nm 'underfocus'
Cs	-7.0×10^{3} nm

C5	2×10^{6} nm
focal spread	0.5 nm
angular spread':	0.1×10^{-3} nm
gaussian spread	2×π×0.33 nm
gaussian spread_range	2×π×0.01 nm
electron dose	5 ×10^{6} e^{-}/ Å^{2}

References

1. G. Kresse, J. Furthmüller, Efficient iterative schemes for ab initio total-energy calculations using a plane-wave basis set. *Phys Rev B Condens Matter Mater Phys* **54** (1996).

2. D. b Kresse G.a Joubert, From ultrasoft pseudopotentials to the projector augmented-wave method. *Phys Rev B Condens Matter Mater Phys* **59** (1999).

3. J. P. Perdew, K. Burke, M. Ernzerhof, Generalized gradient approximation made simple. *Phys Rev Lett* **77** (1996).

4. S. Grimme, Semiempirical GGA-type density functional constructed with a long-range dispersion correction. *J Comput Chem* **27** (2006).

5. D. W. Boukhvalov, M. I. Katsnelson, Destruction of graphene by metal adatoms. *Appl Phys Lett* **95** (2009).

6. T. D. Kühne, M. Iannuzzi, M. Del Ben, V. V. Rybkin, P. Seewald, F. Stein, T. Laino, R. Z. Khaliullin, O. Schütt, F. Schiffmann, D. Golze, J. Wilhelm, S. Chulkov, M. H. Bani-Hashemian, V. Weber, U. Borštnik, M. Taillefumier, A. S. Jakobovits, A. Lazzaro, H. Pabst, T. Müller, R. Schade, M. Guidon, S. Andermatt, N. Holmberg, G. K. Schenter, A. Hehn, A. Bussy, F. Belleflamme, G. Tabacchi, A. Glöß, M. Lass, I. Bethune, C. J. Mundy, C. Plessl, M. Watkins, J. VandeVondele, M. Krack, J. Hutter, CP2K: An electronic structure and molecular dynamics software package -Quickstep: Efficient and accurate electronic structure calculations. [Preprint] (2020). https://doi.org/10.1063/5.0007045.

7. J. Vandevondele, M. Krack, F. Mohamed, M. Parrinello, T. Chassaing, J. Hutter, Quickstep: Fast and accurate density functional calculations using a mixed Gaussian and plane waves approach. *Comput Phys Commun* **167** (2005).

8. J. VandeVondele, J. Hutter, Gaussian basis sets for accurate calculations on molecular systems in gas and condensed phases. *Journal of Chemical Physics* **127** (2007).

9. S. Goedecker, M. Teter, Separable dual-space Gaussian pseudopotentials. *Phys Rev B Condens Matter Mater Phys* **54** (1996).
